# Multi Pro(lo)nged Mechanical Ventilation after Cardiac Surgery

**DOI:** 10.21470/1678-9741-2024-0043

**Published:** 2025-10-31

**Authors:** Rohan Magoon

**Affiliations:** 1 Department of Anaesthesia and Cardiac Anaesthesia, Atal Bihari Vajpayee Institute of Medical Sciences (ABVIMS) and Dr. Ram Manohar Lohia Hospital, Baba Kharak Singh Marg, New Delhi, India. E-mail: rohanmagoon21@gmail.com

To The Editor,

Prolonged mechanical ventilation (PMV) following cardiac surgery continues to be actively researched across diverse clinical settings^[1-3]^. In this regard, a recently published study by Costa AMP et al.^[1]^ retrospectively evaluated the predisposition to PMV and extubation failure in a population of 233 children/adolescents undergoing cardiac surgery. While the authors meticulously assess a range of perioperative factors relating to PMV in their study cohort, their findings need to be interpreted in the light of the following observations^[1-4]^.

It is believed that the wide age gap between 0 and 15 years of the participants renders the study on PMV rather challenging, in addition to introducing a degree of heterogeneity^[1,3]^. For instance, with neonates and infants under a common research frame, an account of prematurity could have been relevant, in addition to presentation of the syndromic patient status by the authors^[2,4]^. A retrospective review by Davis et al.^[2]^, having employed a similar cutoff of 24 mechanical ventilation hours akin to the index study, highlights the role of gestational age in relation to extubation success in children < 36 months who underwent surgery for congenital heart disease (CHD)^[1]^.

Moreover, Tabib et al.^[3]^ outline as to how factors like delayed sternal closure, inotropic use, and vasoactive-inotropic score (VIS) have an independent role to play in predicting PMV in cardiac surgical patients of less than a month to 15 years, as was the case in the Costa AMP et al. study^[1]^. To that effect, a systematic review by Sun et al.^[5]^ associates postoperative VIS with PMV (odds ratio: 5.20; 95% confidence interval: 3.78 - 7.16, *P* < 0.00001, *I^2^* = 41%). Of note, within the 58 studies included in the meta-analysis, only one happened to feature in a non-cardiac setting, with others involving varied cardiac surgical procedures performed across over 29,000 patients^[5]^. Lastly, acknowledging the peculiar research difficulty posed by indigenous postoperative protocols in the PMV studies, future endeavors in the subject would be served well with the reporting on the use of modern-day intraoperative practices such as modified ultrafiltration, especially when evaluating an exclusive CHD patient cohort^[1,6]^.
